# Biomechanical properties of polymer-infiltrated ceramic crowns on one-piece zirconia implants after long-term chewing simulation

**DOI:** 10.1186/s40729-018-0127-5

**Published:** 2018-05-23

**Authors:** Pia Baumgart, Holger Kirsten, Rainer Haak, Constanze Olms

**Affiliations:** 1 Department of Dental Prosthodontics and Materials Science, University of Leipzig, Liebigstraße 12, Haus 1, 04103 Leipzig, Germany; 2 Institute for Medical Informatics, Statistics, and Epidemiology (IMISE), Haertelstraße 16-18, 04107 Leipzig, Germany; 3 LIFE Research Center for Civilization Diseases, University of Leipzig, Philipp-Rosenthal-Straße 27, 04103 Leipzig, Germany; 4 Department of Cariology, Endodontology and Periodontology, University of Leipzig, Liebigstraße 12, Haus 1, 04103 Leipzig, Germany; 5 Department of Dental Prosthodontics and Materials Science, University of Leipzig, Liebigstraße 12, Haus 1, 04103 Leipzig, Germany

**Keywords:** Hybrid ceramic, Polymer-infiltrated ceramic network, PICN, Implant, One-piece, Zirconia

## Abstract

**Background:**

Implant and superstructure provide a complex system, which has to withstand oral conditions. Concerning the brittleness of many ceramics, fractures are a greatly feared issue. Therefore, polymer-infiltrated ceramic networks (PICNs) were developed. Because of its high elastic modulus, the PICN crown on a one-piece zirconia implant might absorb forces to prevent the system from fracturing in order to sustain oral forces. Recommendations for the material of superstructure on zirconia implants are lacking, and only one study investigates PICN crowns on these types of implants.

Accordingly, this study aimed to examine PICN crowns on one-piece zirconia implants regarding bond strength and surface wear after long-term chewing simulation (CS).

**Methods:**

Twenty-five hybrid ceramic crowns (Vita Enamic, Vita Zahnfabrik) were produced using computer-aided design/computer-aided manufacturing (CAD/CAM) technology and adhesively bonded (RelyX™ Ultimate, 3M ESPE) to zirconia implants. Twenty of the specimens underwent simultaneous mechanical loading and thermocycling simulating a 5-year clinical situation (SD Mechatronik GmbH). Wear depth and wear volume, based on X-ray micro-computed tomography volume scans (Skyscan 1172-100-50, Bruker) before and after CS, were evaluated.

All crowns were removed from the implants using a universal testing machine (Z010, Zwick GmbH&Co.KG). Subsequently, luting agent was light microscopically localized (Stemi 2000-C, Zeiss).

With a scanning electron microscope (SEM, Phenom™ G2 pro, Phenom World), the area of abrasion was assessed.

**Results:**

1. After CS, none of the tested crowns were fractured or loosened.
2. The maximum vertical wear after CS was *M* = 0.31 ± 0.04 mm (mean ± standard deviation), and the surface wear was *M* = 0.74 ± 0.23 mm^3^.
3. The pull-off tests revealed a 1.8 times higher bond strength of the control group compared to the experimental group (*t*(23) = 8.69, *p* < 0.001).
4. Luting agent was mostly located in the crowns, not on the implants.
5. The area of abrasion showed avulsion and a rough surface.

**Conclusions:**

PICN on one-piece zirconia implants showed high bond strength and high wear after CS.

## Background

The demand for tooth-colored dental restorations has increased rapidly within the last few years. Ceramic restorations can often meet these requirements. In dental implantology, zirconia especially—due to its esthetical advantage as well as high flexural strength and outstanding biocompatibility—has gained importance [1]. On the other hand, one-piece zirconia implants are not yet commonly used because the surgical possibilities do not always meet the prosthodontics requirements. Besides, angled one-piece zirconia implants are not yet available. The superstructure can only be cemented to the zirconia implant which may result in remaining excess cement and peri-implant inflammation [2].

Implant and superstructure provide a complex system, which has to withstand oral conditions. Concerning the brittleness of many ceramics, fractures are a greatly feared issue. Therefore, PICNs were developed. They are composed of a ceramic and a composite network and are supposed to combine the advantages of both materials [3]. One of these PICN materials is known under the trade name Vita Enamic (VE) (Vita Zahnfabrik, Bad Säckingen, Germany). It consists of 86 wt% feldspathic ceramic and 14 wt% polymer network. The two networks entirely interpenetrate one another which is supposed to result in a high fracture resistance [4].

Low hardness and high fracture stability differentiate PICNs from conventional feldspathic ceramics [5]. Because of a high elastic modulus [6], PICN crowns on one-piece zirconia implants could absorb forces to prevent the system from fracturing when sustaining oral forces. Recommendations for the material of superstructures on zirconia implants are still lacking, and only one study investigates PICN crowns on these types of implants [5].

Accordingly, this study aimed to examine PICN crowns on one-piece zirconia implants regarding bond strength and surface wear after long-term chewing simulation. The number of cycles during chewing simulation (CS) corresponds roughly to an in vivo load of 5 years [7].

## Methods

### Specimen preparation

Twenty-five PICN crowns (Vita Enamic, Vita Zahnfabrik, Bad Säckingen, Germany) for premolars were produced using CAD/CAM technology and polished with the Vita Enamic Polishing Set Technical (Vita Zahnfabrik) as recommended by the manufacturer. All crowns were bonded to identical one-piece zirconia testing implants. The implants were turned from pre-sintered zirconia blocks (VITA In-Ceram® 2000 YZ–55, VITA Zahnfabrik) by the faculty of physics and geosciences at the University of Leipzig. Subsequently, the implants were sintered in a dental laboratory. The abutment had a cone angle of 3 °, while the length of the implant totaled up to 21.5 mm. The abutment length was 6 mm. The thread was conceived schematically.

Twenty of the specimens belonged to the experimental group (*n* = 20) and underwent mechanical loading and wear behavior tests, whereas five of the specimens (*n* = 5) only underwent the pull-off tests.

Five specimens fit into the chewing simulator which is why five specimens were prepared at a time. Therefore, four rounds of CS were performed.

All steps of the bonding procedure followed the manufacturer’s instructions: the bonding surface of the crown was degreased with alcohol and conditioned with 5 % hydrofluoric acid gel for 60 s (Vita Ceramics Etch, Vita Zahnfabrik). The hydrofluoric acid gel was removed with water spray and the bonding surface was dried for 20 s. Conditioning of the bonding surface of the implant was ensured by sandblasting with aluminum oxide (Al_2_O_3_) 110 μm at 1 bar and cleaning with alcohol. After that, a bonding agent (Scotchbond Universal, 3M ESPE, St. Paul, MN, USA) was applied to the surfaces to bond the crown and the implant and both dried with air. The crowns were adhesively bonded (RelyX™ Ultimate, 3M ESPE) to the one-piece zirconia implants. Photopolymerization of the luting agent was carried out by a dental curing light for 40 s on each surface.

**Fig. 1:**
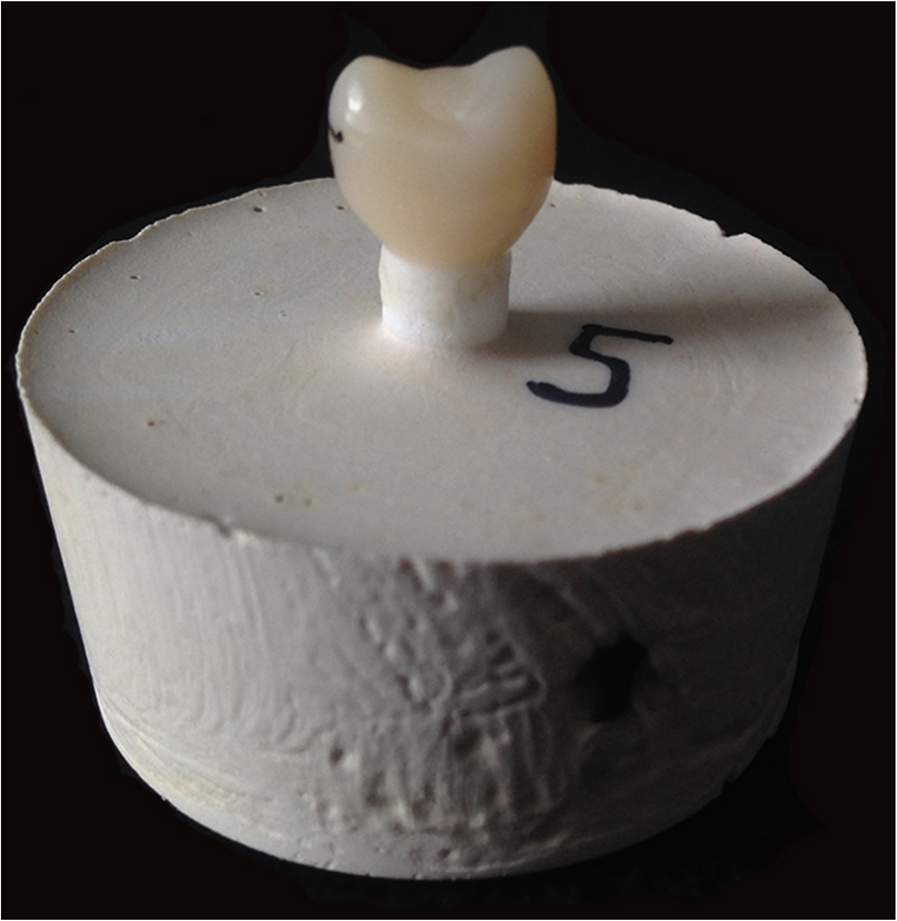
Luted crown on embedded implant before chewing simulation

All specimens were embedded in acrylic resin (Technovit 4000, Heraeus Kulzer GmbH, Wehrheim, Germany) with a parallelometer for the exact vertical orientation. Epoxy was prepared according to manufacturer’s data, and the specimens were embedded directly into the sample holder of the chewing simulator. Figure 1 shows a luted crown on an embedded implant ready for CS.

The specimens attached to the parallelometer were perpendicularly recessed until only the upper coils of the implants were on view.

To produce replicas of the specimens from the experimental group, the crowns’ occlusal was cast using VPS Hydro Putty und VPS Hydro Light Body (Henry Schein Inc., New York, USA) before and after CS. The impression was grouted with Stycast 1266 (Loctite Henkel Electronic Materials, Westerlo, Belgium). The replicas could be scanned by X-ray micro-computed tomography (Micro-CT, Skyscan 1172-100-50, Bruker microCT, Kontich, Belgium). Table 1 shows the scanning parameters of the replicas before and after CS.

### Chewing simulation

The specimens of the experimental group underwent long-term chewing simulation (SD Mechatronik GmbH, Feldkirchen-Westerham, Germany): 1,200,000 cycles, 50 N, and simultaneous thermocycling of 5500 cycles with changing temperatures of 4 and 56 °C for 60 s each. Hydroxyapatite steatite indenters (6.35 mm diameter) were used as antagonists and were replaced for each specimen. The indenter slid 1.5 mm down the inner cliff of the vestibular cusp and 0.5 mm horizontally toward the central fossa at a speed of 60 mm/s each. Five specimens underwent CS at the same time.

The specimens from the control group did not undergo CS. Failure was defined as fracture within the system (crown or implant) or loosening of the crowns during or after CS.

### Wear behavior after long-term mechanical loading

**Fig. 2:**
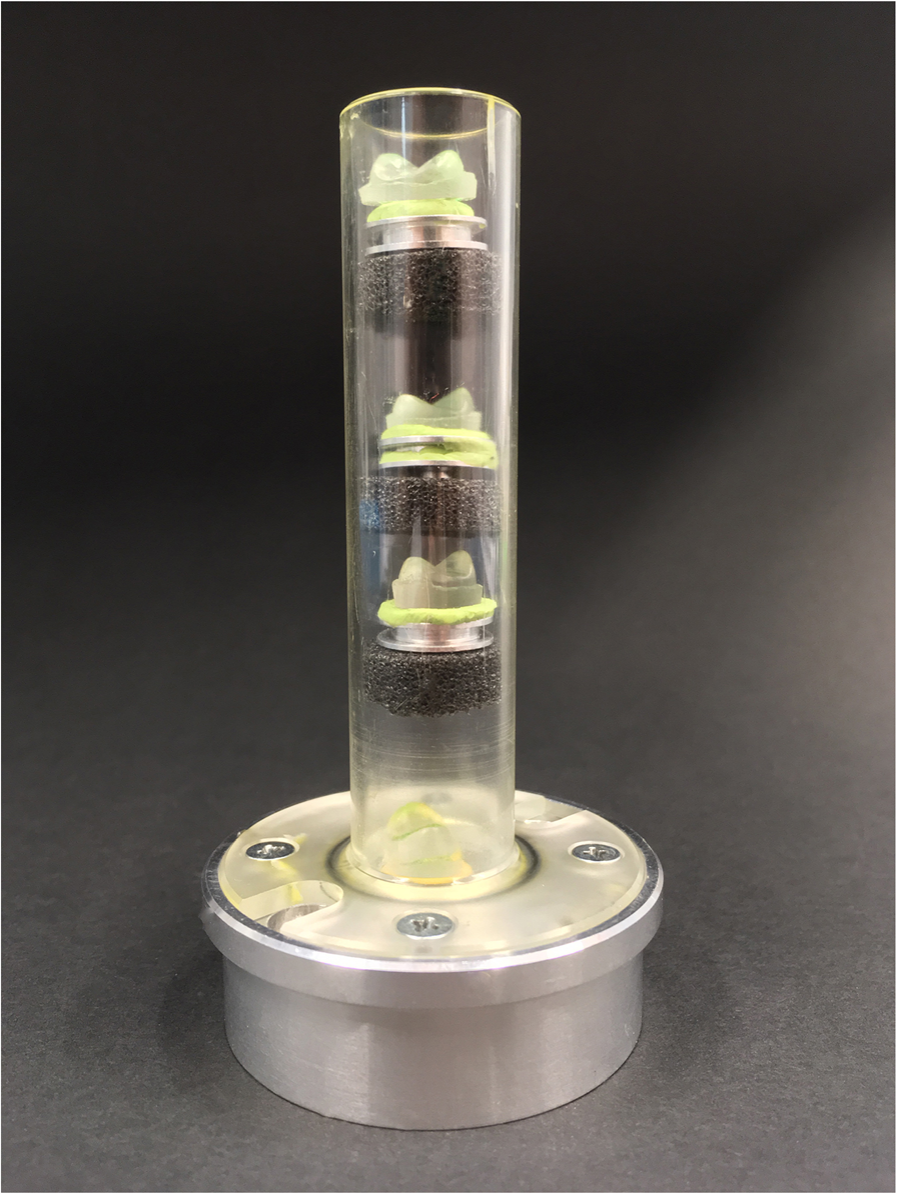
Four replicas on specimen stubs and foam pellets in the sample holder of the Micro-CT

After CS, replicas were produced in the same way as before CS. A commercially available dough, aluminum holder (SEM Specimen Stubs, Agar Scientific, Essex, UK), and foam pellets allowed four replicas to be attached at the same time to the tubes of the Micro-CT (Fig. 2). One single specimen could not be scanned due to a mistake during grouting.

For the generation of 3D data sets from the scans of the Micro-CT, the program NRecon v.1.6.10.4 (Bruker microCT) was employed. The software could reduce ring artifacts by 20 (Ring Artifact Correction). Beam hardening correction was set to 60 %.

For volume assessment of abrasion, each 3D data set was segmented before and after CS in CTAn (CTAnalyzer V.1.15.4.0, Bruker microCT). Both data sets were overlapped, and the remaining volume of abrasion quantified in pixels and converted into cubic millimeters.

The maximum wear depth was determined by “blowing up” virtual bullets within the surface of abrasion. The diameter of the most massive bullet (at the spot of maximum wear depth) was measured in pixels and converted into millimeters.

**Fig. 3:**
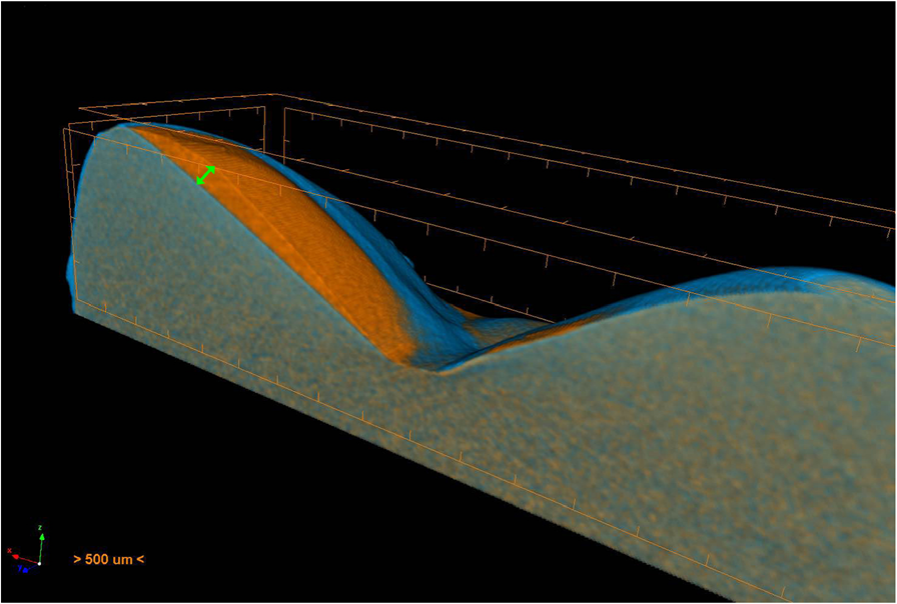
Area of abrasion (yellow surface) and maximum vertical wear (arrow)

The arrow in Fig. 3 shows the maximum wear depth after CS. Volume wear is demonstrated as a yellow surface. Descriptive statistical analysis was applied.

In addition to quantifying wear behavior, one specimen from the test group was randomly selected for analyzing qualitative wear behavior with a scanning electron microscope (SEM, REM, Phenom™ G2 pro, Phenom-World). Before SEM imaging, the crown was gold-coated (2 nm, Sputter Coater MSC1, Ingenieurbüro Peter Liebscher, Wetzlar, Germany) to prevent accumulation of electrostatic charge.

### Pull-out forces and localization of luting agent

The crowns were removed from the implants using a universal testing machine (Z010, Zwick GmbH&Co.KG, Ulm, Germany). To do so, the embedded specimens (crown + implant) were placed in a specially built device and covered with a base metal alloy which was specially created as well. A preload of 1 N was applied vertically to the crown followed by traction of 0.75 mm/min. Load at breakage/removal was recorded. The bond strength from the specimens from both the control group without CS (*n* = 5) and the experimental group after CS (*n* = 20) was measured.

Luting agents on both the crown and the implant after CS were localized under a stereomicroscope (Stemi 2000-C, Zeiss, Karlsruhe, Germany). Representative pictures of each crown and implant were taken, and a percentage of luting agent on crown and implant was recorded descriptively.

### Statistical analysis

The statistical analyses were performed using GNU Project (2015) (GNU PSPP (Version 0.8.5) [Computer Software]. Free Software Foundation. Boston, MA). The Kolmogorov-Smirnov test, visual inspection of the distribution of the data in histograms as well as in quantile-quantile plots, was applied to verify if the data were normally distributed. The ANOVA test was used to analyze the differences in the mean level of the four rounds of CS concerning bond strength, maximum vertical wear, and volume wear of the experimental groups. A *t*-test for independent samples was performed to find differences in bond strength between the experimental and the control group. Student’s *t*-test was applied assuming no different variants between control and experimental group as no empirical difference of the variances was observed (*p* = 0.755, Levene’s test). The exact confidence interval was calculated according to Clopper-Pearson.

**Table 1:** Micro-CT scanning parameters of the replicas before and after CS

Voltage	60 kV
Amperage	167 μA
Filter	No filter
Angle step	0.7°
Scanning resolution	Large pixel scan, 960 × 666 pixels
Rotation angle	180 °
Voxel size	14.985 μm
Frame averaging	20
Random shift	10

## Results

**Fig. 4:**
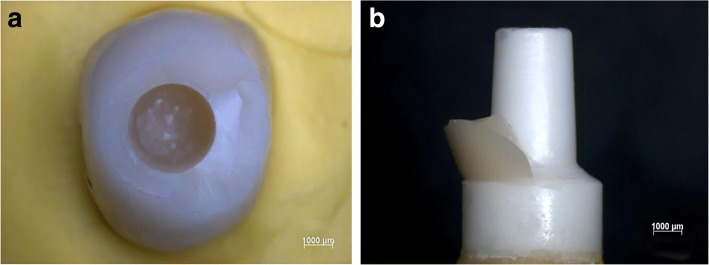
Luting agent located mostly in the crown (**a**) and only sparsely on the implant (**b**). A crown fragment is remaining on the implant

**Fig. 5:**
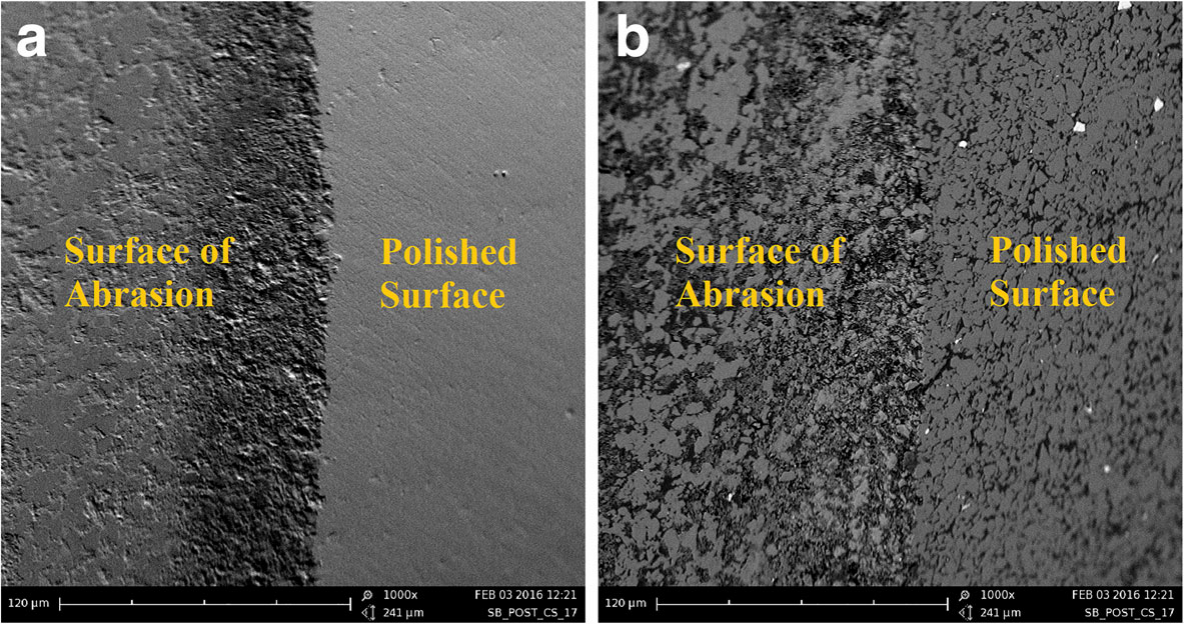
SEM images of the mesial margin of abrasion under topography contrast (**a**) and material contrast (**b**)

1. No failure occurred as none of the tested crowns or implants was fractured or loosened during or after CS.
2. The tested crowns showed a maximum wear depth of *M* = 0.31 ± 0.04 mm (mean ± SD) and volume wear of *M* = 0.74 ± 0.23 mm^3^ (mean ± SD). Table 2 shows the mean and standard deviation of assessed parameters (pull-out forces, maximum wear, volume wear) of each round of CS. Abrasion was macroscopically observed.
  The Kolmogorov-Smirnov test and a visual inspection of the histograms and the quantile-quantile plot showed no significant divergence from the normal distribution in any of the groups (maximum wear depth after CS, volume wear after CS, pull-off forces without and after CS).
3. One-way ANOVA showed differences neither in pull-out forces *F*(3,16) = 0.02, *p* = 0.997, nor in maximum wear *F*(3,15) = 0.39, *p* = 0.764, or volume wear *F*(3,15) = 0.77, *p* = 0.530, among the four rounds of CS (Table 3), thereby demonstrating stable and comparable conditions within all rounds of CS.
4. In the pull-out tests, the crowns from the control group were removed from the implants at a 1.8 times higher load (*M* = 588.4 ± 57.7 N) than the crowns of the experimental group (*M* = 322.8 ± 61.9 N). Therefore, the bond strength of the control group was significantly higher than the bond strength of the experimental group (*t*(23) *=* 8.69, *p* < 0.001). Table 4 shows the resulting characteristics of PICN crowns on one-piece zirconia implants.
5. Under the stereomicroscope, approximately 90% of the luting agent could be stereomicroscopically located in the crowns, not on the implants. Figure 4 shows the luting agent situated mostly in the crown (a) and only sparsely on the implant (b).
6. The crowns' surface of abrasion revealed avulsion and a rough surface under SEM. The polished surface and the surface of abrasion do not appear similar. Figure 5 shows SEM images of the mesial margin of abrasion under topography (a) and material contrast (b).

**Table 2:** Mean (standard deviation) of assessed parameters

CS round (*n*)	Pull-out forces	Maximum wear	Volume wear
#1 (5)	319.6 (75.4)	0.33 (0.05)	0.88 (0.31)
#2 (5)	326.2 (75.0)	0.30 (0.04)	0.71 (0.20)
#3 (5)	319.4 (43.9)	0.32 (0.03)	0.68 (0.18)
#4 (5)	325.9 (69.8)	0.31 (0.07)*	0.69 (0.27)*

*Only four specimens could be analyzed due to a mistake during grouting *n* number of samples per round

**Table 3:** Stability of conditions across four CS rounds

ANOVA results	Pull-out forces	Maximum wear	Volume wear
*F* (df)	0.02 (3, 16)	0.39 (3, 15)	0.77 (3, 15)
*p* value	0.997	0.764	0.530

No statistically significant differences were observed between rounds (testing the null hypothesis that means are similar across all four rounds of CS). This supports stability and comparability of the experiments

**Table 4:** Characteristics of polymer-infiltrated ceramic crowns on one-piece zirconia implants

Characteristics	Total *n*	Observations
With CS		
System fractured	20	0% (95% CI 0–16.8%)
Crowns loosened	20	0% (95% CI 0–16.8%)
Maximum wear depth	19	0.31 mm (0.04 mm)
Volume wear	19	0.74 mm^3^ (0.23 mm^3^)
Bond strength (pull-out test)	20	322.8 N (61.9 N)*
Without CS		
Bond strength (pull-out test)	5	588.4 N (57.7 N)*

If not stated otherwise, means (standard deviations) of assessed parameters are shown

*p < 0.001 for comparing the effect of performing a CS (experimental group) vs. performing no CS (control group) on bond strength according to the null hypothesis of no difference between both groups

## Discussion

To the best of our knowledge, it was the first time that the biomechanical properties of polymer-infiltrated ceramic crowns on one-piece zirconia implants after long-term chewing simulation were examined. The present in vitro study investigated the biomechanical properties concerning surface wear and bond strength. No fractures occurred during long-term chewing simulation, and the abrasion of the crowns was macroscopically visible. There are several reasons for the fracture resistance:

Firstly, the layer thickness prescribed by the manufacturer could be strictly adhered to.

Due to the sizes of the probational implants, enough friction surfaces on the implants could be ensured and fracture and debonding was less likely.

Lastly, the occlusal force of 50 N appointed in the chewing simulator is distinctly lower than the maximum in vivo bite force of approximately 700 N [8]. 50 N roughly imitates light biting [9].

El Zhawi et al. also investigated wear and fatigue fracture of PICN crowns (Vita Enamic) but attached to composite abutments instead of zirconia implants [10]. They tested VE crowns after long- and short-term biomechanical loading. The specimens from the long-term mechanical loading group, which are most likely to be compared to our study, did not undergo any pull-off tests. In both studies, no failure occurred during or after mechanical loading. A remarkable difference between the results of both studies was seen in surface wear of the crowns which was much higher in our study despite the lower load of 50 N instead of 200 N. The materials’ characteristics may explain the relatively high wear of the crowns. Compared to composites and dentin-like materials, zirconia is a very rigid material. During chewing simulation, the implant does not move so there is only one component of the system to absorb the occlusal force which may result in high wear. Even though surface wear was macroscopically visible, abrasion may also prevent the system from catastrophic failure, namely, fractures in the implant.

In the study of Naumova et al., volume and vertical wear of PICN crowns, compared to other materials such as a nanoceramic resin and a lithium silicate reinforced ceramic after CS, were tested [11]. They used the same settings of CS as in the present study, but the crowns were luted to extracted molars instead of implants and extracted molars as antagonists were used as well. Concerning volume and vertical wear after CS, PICN crowns showed the lowest cusp abrasion, much lower than in our study. Due to the use of abutment teeth instead of dental implants, the results cannot be compared to ours.

Mörmann et al. compared the surface wear of different dental ceramics including Vita Enamic [12] after the same type of mechanical loading as in the present study. The results showed similar wear to other CAD/CAM materials as well as to human enamel. Since enamel of extracted molars was used as indenters in this study, it cannot be compared directly to our procedure. Nevertheless, a remarkable difference could be found in the results of the SEM images. Mörmann et al. described the surface of abrasion as similar to the polished surface [12], which cannot be found in our specimen.

According to Lauvahutanon et al., PICN crowns show minor wear compared to direct restorations made of composite [13].

Until now, there have been numerous publications on VE [3–6, 10–24] but only one study has investigated the combination with zirconia implants [5] where fracture strength of VE and feldspathic ceramic on zirconia implants was compared using different luting agents. Fracture strength was tested by applying an axial force to the specimen until fracture. The results showed higher fracture strength of VE. The samples were placed in distilled water for 24 h after cementing, so no dynamic loading occurred. Due to the different study designs—no pull-off forces, no dynamic loading, and no wear tests—it cannot be compared to ours.

The missing comparison to other PICN materials can be considered a limitation of the study. Since VE is a unicum in the family of PICN materials, it is difficult to find an appropriate material of comparison, especially since Lava Ultimate (3M Espe), a resin nanoceramic, is no longer indicated as a crown material due to a high rate of loosening. The review of Mainjot et al. reported that the loosening has mostly occurred when bonded to zirconia and that there is a lack of studies concerning bonding of VE to other ceramics [25].

Although the sample size of this pilot study is limited (due to the practicability reasons associated with the applied procedures), the standard deviations are low, which improved the statistical power of our analysis.

Surface wear of replicas of the superstructure’s occlusal was assessed by Micro-CT instead of the crowns themselves. This was done to entrench the Micro-CT as a clinical method to quantify abrasion. Using a Micro-CT for quantifying abrasion could be a non-invasive option without radiation exposure for the patient. Additionally, the grouting material (Stycast Epoxidharz) exhibits a very low viscosity of 0.65 Pa s [26] and therefore a high flowability even in small volumes which can result in exact replicas.

The use of spherical steatite indenter during CS instead of natural teeth with their anatomy and composition may be a limitation of the study.

Abrasion may depend on the type of construction as well. Wear of VE crowns on one-piece zirconia implants seems different from wear of VE crowns on dentin-like materials [10]. This aspect should be investigated in further studies.

## Conclusions

The present study demonstrates that elastic PICN crowns on rigid one-piece zirconia implants seem to be a promising material combination for clinical practice. Though the crowns suffered major wear after CS, the stability was not affected, and no catastrophic failure occurred. However, clinical trials are essential to examine the behavior of the material combination, especially in comparison to other restorative materials.

Micro-CT for replicas proved to be able to measure surface wear of dental restorations.

## Abbreviations

3D: Three-dimensional space
ANOVA: Analysis of variance
CAD/CAM: Computer-aided design/computer-aided manufacturing
CI: confidence interval (exact) according to Clopper-Pearson
CS: Chewing simulation
df: degrees of freedom
et al.: Et alii/et aliae/et alia
*F*: *F* test
M: Mean
Micro-CT: X-ray micro-computed tomography
*n*: Number
*p*: *p* value
PICN: Polymer-infiltrated ceramic network
SD: Standard deviation
SEM: Scanning electron microscope
VE: Vita Enamic
